# Practice and experience in the teaching system of clinical pharmacy laboratory in the post-epidemic era: A review

**DOI:** 10.1097/MD.0000000000032223

**Published:** 2022-12-09

**Authors:** Qi Huang, Hong Su, Yingfan Zhang, Shao Liu, Qiong Liu, Yueping Jiang

**Affiliations:** a Department of Pharmacy, Xiangya Hospital, Central South University, Changsha, China; b Department of Pharmacy, Xiangya Changde Hospital, Changde, China; c Operating Room, Xiangya Hospital Central South University, Changsha, China; d Department of Oncology, Xiangya Hospital of Central South University, Changsha, China.

**Keywords:** biosafety, clinical pharmacy, post-epidemic era, progressive teaching, TDM

## Abstract

Out of the global outbreak of COVID-19, clinical pharmaceutical therapeutic analytical-teaching laboratories underwent an increasing number of digitally-led teaching research. A teaching system working online and offline to monitor medicinal drugs was explored and established using a clinical pharmaceutical therapy through a drug concentration monitoring laboratory within a comprehensive tertiary hospital. Meanwhile, laboratory access training and standards of laboratory biosafety management system were also strictly implemented, improving the technical operation and daily management. Moreover, a new, significant, and efficient teaching mode was set up based on vocational training needs for efficient and professional learning. The learning results are enforced to have dynamic checks accomplished using stage-oriented assessment. Moreover, the questionnaire survey results, especially during independent learning ability and laboratory skills training, reveal that teachers and students have commented positively on the new teaching mode. Hereon, a clinical pharmaceutical teaching system during the Post-Epidemic Era was elaborated to provide a unique teaching mode and experience dedicated to teaching and scientific research in clinical therapeutic drug monitoring laboratory.

## 1. Introduction

Therapeutic Drug Monitoring (TDM) is a vital analytical technology for the drugs in the hospital clinical pharmacy. Some substances in the blood sample of a patient can be detected using modern analytical detection methods, including drug exposure, pharmacological markers, and Post-Epidemic Era pharmacodynamics indicators. Meanwhile, as a baseline for drug therapy window and used in the quantitative pharmacological model, which improved the individualized administration for patients. In the Post-Epidemic Era, drug concentration monitoring technology of antiviral drugs was actively developed by Lab pharmacists to explore the clinical significance of TDM, playing a positive role in promoting the progress of clinical research on COVID-19.^[1-3]^

Nowadays, precision medicine has become the mainstream mode of modern medicine.^[4]^ Domestic medical institutions at all levels have successively developed a clinical pharmaceutical treatment using drug concentration monitoring laboratories (hereinafter referred to as “laboratories”). This is used for personalized clinical medicine and the teaching work of laboratory pharmacists. Our research group teaches TDM to interns, graduates, and advanced students. The teachers arranged entire offline theory and operation courses for students, guiding students to undergo the methodological establishment and verifying the new testing items.

In the Post-Epidemic Era, laboratory pharmacists must take good personal protection and enhance biosafety awareness. Meanwhile, they should develop safer and more efficient tests and conduct clinical studies through blood concentration monitoring technology. However, these changes require high professionalism, and the teaching quality of TDM teaching institutions plays a decisive role in adapting to the new situation. In addition, offline experiment hours have reduced sharply, and part of some professional laboratories underwent online teaching during the epidemic period.^[5-9]^ Therefore, based on the national epidemic prevention and control requirements and the deficiencies within the existing teaching system, our research group is attempting to improve the teaching work of the laboratory by sending questionnaires to teachers and students to evaluate the overall improvement effect from 2021 to 2022.

## 2. Methods

The research group collected feedback on the teaching work from teachers and students after assessing the 2020 to 2021 academic year. Some students thought they only completed the daily testing work and could not understand the working principle behind the analytical instruments or the application of the test results to the clinical treatment during the internship. Due to the epidemic, some teachers said it was difficult to carry out their teaching plans without enough opportunities to carry out face-to-face teaching. In addition, the leading daily work of the laboratory is to process blood samples. Current studies have established that SARS-COV-2 RNA could be detected in the blood of COVID-19 patients and transmitted as aerosols.^[10,11]^ However, some operations such as centrifugation, vortices, and pipetting in the pretreatment of blood samples can produce aerosols. Blood samples with the virus are a potential source of infection among laboratory workers. Therefore, besides daily epidemic prevention and control, teachers and students should undergo adequate biosafety training, and experimental operations should be standardized. Through laboratory adjustment for teacher structure, biosafety was accepted as the key content of laboratory access training, optimizing the teaching system and evaluation method, and improving the teaching work during the 2021 to 2022 academic year (Table 1).

**Table 1:** Comparison between traditional teaching and post-epidemic teaching.

Content	Traditional teaching	Teaching in the post-epidemic era
Tutor system	Single tutor system	Dual tutor system
Laboratory access training	Face to face teaching	Self-learning + tutorial
Teaching model	Full guidance	Progressive teaching
Evaluation method	Traditional final examination	Periodic assessment

### 2.1. “TDM Pharmacist + specialist clinical pharmacist” dual tutor system

The medical institution of our laboratory has high-quality teachers and significant teaching resources. Combined with the teaching needs, clinical pharmacists from the Department of Pharmacy were introduced to participate in TDM teaching, forming the “TDM pharmacist + specialist clinical pharmacist” dual tutor system unique to our department. Each teacher has an intermediate professional title after professional level, teaching ability, and ideological and moral assessment layer upon layer of selection. Each teacher has an intermediate professional title and has passed the biological safety training organized by the medical institution, according to the research direction, divided into TDM teachers and clinical pharmacy teachers. For instance, TDM teachers possess significant working experience, mainly responsible for laboratory teaching, scientific research thinking training, and graduation design. Specialized clinical pharmacy teachers have several years of clinical work experience, supervising students to participate in clinical practice, cultivating clinical thinking, and guiding students to participate in clinical research with TDM teachers. The teachers and students formed teaching groups based on the 2-way wishes. Each group had 1 TDM and 1 clinical pharmacy teacher having the same research direction. The number of students within each group was 4 to 6.

### 2.2. Laboratory access training focusing on biosafety

The U.S. Centers for Disease Control and Prevention advises that any procedure generating aerosols or droplets should be performed in a certified level 2 biosafety cabinet.^[12]^ Our laboratory underwent a laboratory-associated infection risk assessment following the World Health Organization (WHO) Laboratory Biosafety Guidelines for COVID-19 to safely perform the intended tests with appropriate risk prevention and overall control measures.

Liu recommends that laboratory personnel without biosafety training should not be allowed to work in laboratories during the COVID-19 pandemic.^[13]^ We extended the laboratory access training from 2 weeks to 1 month. The theoretical content mainly focuses on independent learning of the “Laboratory Safety Education Manual” and video courses and helps them improve their practical operation skills through on-site training. Meanwhile, the manual lists biosafety-related systems and operating procedures as essential learning content and the video courses have been arranged and recorded by the laboratory teachers (Table 2).

**Table 2:** Video course arrangement for the laboratory access training.

Course type	Course title	Class time
Teaching introduction	What is therapeutic drug monitoring?	1
	Introduction to the laboratory environment, instruments, and equipment	2
Test content	Sample pretreatment	3
	Injection analysis and data processing	2
	Interpretation of test results	1
Biosafety	Epidemiological characteristics of novel Coronavirus	1
	Laboratory and Novel Coronavirus	1
	Daily biosafety protection requirements	2
	How to wear and remove biosafety level 2/3 PPE correctly?	1
	Biosafety practice for blood Samples from confirmed/suspected COVID-19 patients	2
	Handling errors and accidents	2
	The medical waste disposal process	1
	Laboratory clearance and disinfection process	1

The on-site training has been uniformly arranged in the afternoon. Teachers of each group decided the order of entering the laboratory through drawing lots in advance. The group training can ensure that each student has enough opportunities to get started. Each group of students is fixed in the same laboratory for follow-up study and research to reduce staff mobility. Teachers and students should communicate frequently and process blood samples in actual study and life. Corresponding requirements have been made for equipment management, such as at least a 1-meter distance between laboratory seats, keeping the table clean before and after the experiment, placing instruments and equipment independently, and forming a biosafety management system:

The health code and travel code should be displayed before entering the laboratory, which is a green pass.

In high-risk period, students should provide negative nucleic acid test certificate every week, which the teachers will collect.

Medical protective masks and work clothes should be worn after entering the laboratory, along with level ii/III personal protective equipment based on the specific work situation while handling blood samples.

Frequent walking and entering the laboratory should be avoided.

Online communication through QQ, WeChat and other social software should be encouraged. If face-to-face communication is required, crowd gathering should be avoided, and a social distance of at least 1 meter must be kept.

Hand hygiene should strictly follow the requirements of “seven-step washing techniques.”

After the operation, the working area should be cleaned and disinfected.

Students must participate in the theoretical and practical operation assessment post-training. Theoretical examination topics are from the contents of the manual, primarily to investigate the knowledge of SARS-COV-2-related microbiology, infectious diseases, epidemiology, infection control, and other relevant technical laboratory specifications, standard operating procedures, and biosafety protection. The assessment of practical operation includes biosafety level-II/level-III personal protective equipment wearing and the unloading process, laboratory operation of simulated blood samples from confirmed or suspected COVID patients, and emergency drill. The assessment criteria can be found within the supplemental content (See Tables S1, http://links.lww.com/MD/I94 and S2, http://links.lww.com/MD/I95, Supplemental Content, Table S1, http://links.lww.com/MD/I94 illustrates the clinical pharmacy laboratory biosafety level II/III personal protective equipment wear and doff operation assessment standards. Table S2, http://links.lww.com/MD/I95 illustrates the laboratory assessment standards for simulating blood samples from patients with confirmed or suspected COVID). Students with scores higher than 80 could participate in the follow-up study. For those failing to reach the standard, their time will be extended to participate in the laboratory access training

### 2.3. “progressive” teaching system

Interdisciplinary team collaboration has become a novel strategy to improve the quality of medical care.^[14]^ Thus, establishing a teaching mode that focuses on cultivating clinical thinking and scientific research innovation ability among students can enable compound TDM pharmacy with innovation and application to adapt to the new medical mode.

The laboratory combines various teaching modes with the characteristics of the epidemic period, developing a unique “progressive” teaching system within the laboratory. The form of theoretical teaching was changed from face-to-face to online, and teachers utilized the Tencent conference software to give lectures to students. Despite the disadvantage of online education, the general effect is no better than offline. Since it has just stepped into the lab, students lack the TDM system to acquire theoretical knowledge. They need to obtain an adequate theoretical basis to adapt to the gradually in-depth tasks quickly. Meanwhile, online teaching can effectively avoid the gathered students to minimize the spread of the epidemic.

Students should strictly abide by the requirements of the laboratory biosafety management system, and teachers will gradually modify the teaching process form from lecture-oriented to question-based. The initial questions, such as the methods involved in blood sample pretreatment, were relatively simple. These questions will deepen as the research progresses. Therefore, students must summarize and organize by themselves after consulting professional textbooks and literature and share their learnings through weekly online group meetings, and their teacher would not answer these questions directly. The students understand that TDM sample pretreatment methods include protein precipitation, liquid-liquid extraction, and solid phase extraction. However, their drawback is that the procedures are cumbersome. Recently, a new pretreatment technology using direct injection has emerged.^[15]^ The advantage is that it can simplify operation steps, effectively reducing the generation of aerosols. Under the epidemic prevention and control policies, the teachers’ unions will organize offline meetings. In addition to face-to-face coaching, moreover, teachers can provide online guidance through QQ, WeChat, and other social software to broaden their research ideas when students encounter complex problems during independent project research.

The teaching of clinical theoretical knowledge is mainly case teaching. Specialized clinical pharmacy teachers select typical clinical cases and design questions for students. Each student independently needs to consult literature and books and summarize their knowledge through group discussion. Specialty clinical pharmacy teachers regularly organize students to participate in clinical practice such as pharmaceutical ward rounds, medication education, and sending test reports to deepen their understanding and truly integrate clinical knowledge within the TDM work.

### 2.4. Periodic assessment

In this experiment, the content of the assessment was adjusted. A comprehensive evaluation was carried out from 3 aspects: learning report, final theory examination, and regular performance. The final assessment was calculated following the score ratio of 6:3:1. We reduced the proportion of theoretical test scores in the total score and carried out periodic monthly evaluations of students. During the group meeting in the fourth week of each month, students would make regular learning reports through PowerPoint (protein precipitation), including reading reports, case discussions, project research progress, etc, and devise plans for the next month. Teachers grade students’ reports using 6 aspects: content completeness, learning completion, research rationality, clinical thinking, scientific research innovation ability, and expression ability. The score of each item was 10 points, and the reported scores of each stage were the average scores of TDM teachers and specialist clinical pharmacy teachers. The part of the final score calculation for (Sum of learning report score at each stage/times of participating in the report) × 40%+ Final report score × 60%.

Theory examination was carried out over the internet, and the system randomly selected questions from the question bank. A biosafety-related content was added to the question bank, and the questions with low relevance to the teaching content were eliminated. The total number of questions was reduced to 60 from 100. The students’ political thought, labor discipline, learning attitude, and compliance using the laboratory biosafety system will be evaluated monthly by the TDM teachers.

## 3. Result

After the assessment, 62 students (8 interns, 40 graduates, and 14 visiting students) and 12 teachers were given anonymous and nonpublic questionnaires to evaluate the teaching improvement effect (Table 3). For questions 7 to 20 on the scale, teachers and students must choose from the 5 options with a score of 1 to 5 based on their agreement with the items (teachers only need to answer questions 7–10). The non-scale questions are mainly used to collect students’ basic information. We abandoned the traditional open questions to get more teachers and students’ suggestions on teaching improvement. We adopted the semi-open questions (questions 21 and 22), combining multiple choice and short answers to collect the information.

**Table 3:** Contents of the questionnaire.

Item	Question
1	Your gender
	• Male
	• Female
2	your academic degree
	• Undergraduate
	• Postgraduate and above
3	your major
	• Clinical pharmacy
	• Pharmacy
	• Other pharmacies (fill in the blanks)
4	The student body to which you belong
	• Intern
	• Postgraduate
	• Advanced student
5	Your participation in the new test development progress of TDM
	• Methodological establishment
	• Methodological validation
	• Completed
	• Project approval
	• not involved
6	Are you involved in any research related to TDM
	• Yes
	• No
**Evaluation of the teaching improvement effect by the teachers and students**	
The following teaching jobs do you prefer	
7	Tutor system*^,^†
8	Laboratory access training*^,^†
9	Teaching model*^,^†
10	Evaluation method*^,^†
**Questionnaire questions related to biosafety training**	
11	Through laboratory admission training, your biosecurity awareness is enhanced*
12	You can follow the daily biosafety requirements of the laboratory strictly
13	In the event of a failure or an accident that raises a biosafety issue, you will be able to deal with it accurately*
**Question related to special teaching methods during the pandemic**	
14	The process of video self-learning experiment makes you feel boring*
15	When experimental research is blocked, teachers can provide satisfactory experimental guidance online*
16	You prefer to have face-to-face discussions with teachers and classmates in the lab rather than in an online group*
**Questions related to the improvement of professional competence**	
You think your professional ability to improve the degree after the training	
17	Professional theory*
18	Experimental operation*
19	Clinical thinking*
20	Scientific thinking*
21	What do you think are the advantages of training‡
22	Do you think there are deficiencies in training‡

TDM = therapeutic drug monitoring.

* 5-point Likert scale questions

† Questions contained in the teacher questionnaire

‡ Semi-open-ended questions

Thirty-eight valid questionnaires were obtained from students (8 interns, 22 graduate students, and 8 advanced students) and 12 useful questionnaires from teachers, with recovery rates of 61.3% and 100%. Among the 38 students, 12 had a bachelor’s degree, and 26 had a master’s degree or above. There were 10 clinical pharmacy and 28 non-clinical pharmacy majors. The results of the questionnaire are depicted below.

### 3.1. Teachers and students’ tendentious evaluation of teaching system

**Figure 1.**
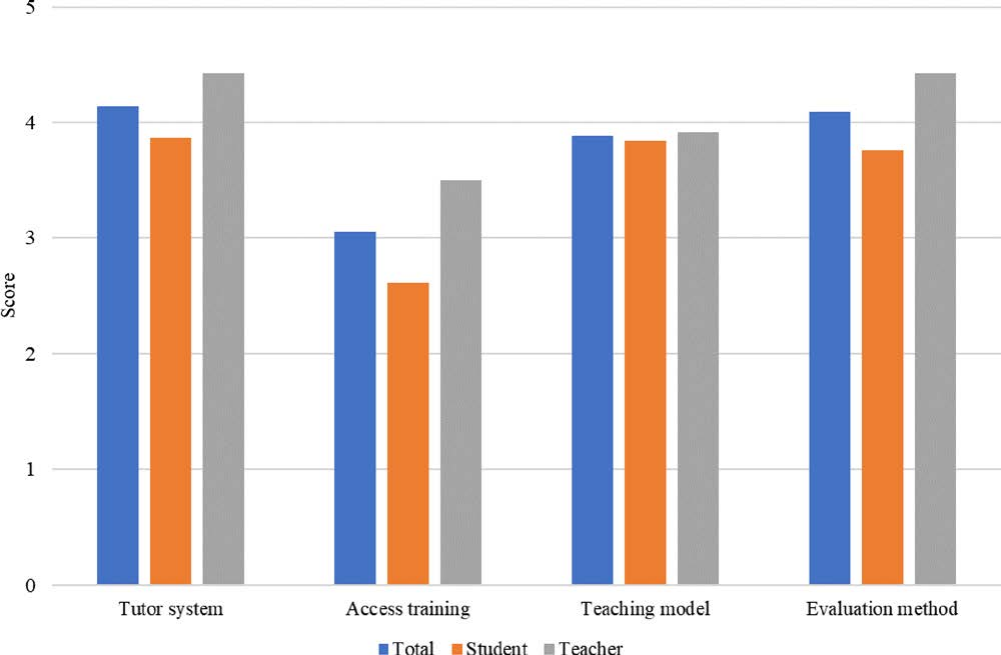
Teachers and students’ tendency evaluation of the teaching form. Numerical value is the average of the analyzed items. No significative differences were found between degrees.

In questions 7 to 10, teachers and students need to rate the improvement effect of the tutor system, access training, teaching model, and evaluation method. The higher the score is, the more inclined they are to the improved teaching mode. The survey results indicate that teachers and students positively evaluate teaching improvement work (Fig. 1). Although they have different opinions regarding laboratory admission training, the data differences are incomparable.

**Figure 2.**
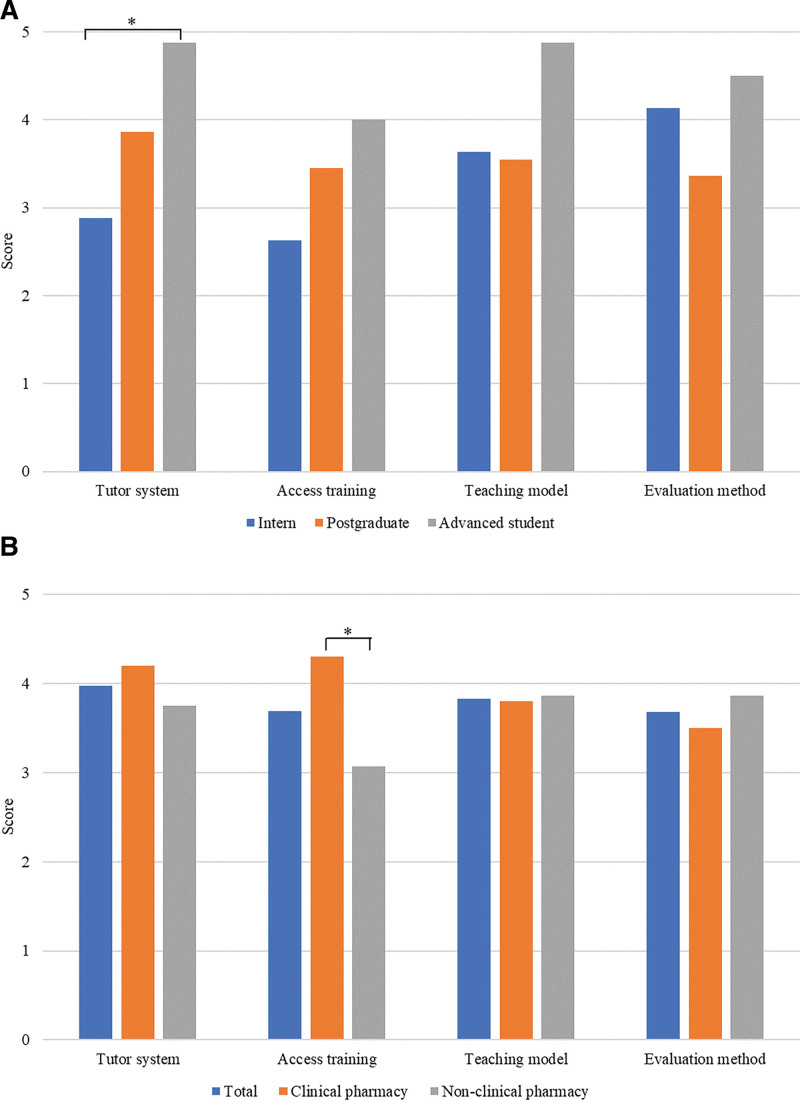
Orientation evaluation of the teaching forms of students from different groups and majors. (A) Evaluation of the orientation of different student groups through the teaching form. (B) Evaluation of students’ preference through teaching forms in different majors. The values have been expressed as the average of the analyzed items. All the data underwent the normal distribution and homogeneity test of variance. Independent sample *t* test and 1-way analysis of variance were used to compare the different samples, *P < .05.

Compared with interns, advanced students become inclined toward the dual-tutor system (4.88 vs 2.88, *P* < .05) (Fig. 2A). On the one hand, they need to improve clinical thinking by learning to meet the needs of future work and research. The addition of specialist clinical pharmacy teachers could enrich clinical theory and practice teaching content. On the other hand, interns lack working experience in hospital pharmacy without realizing the importance of clinical knowledge during TDM practice. Students majoring in clinical pharmacy became more adaptable to self-taught laboratory access training than those majoring in non-clinical pharmacy (4.30 vs 3.07, *P* < .05) (Fig. 2B). It could be because traditional pharmacy majors do not understand personalized drug therapy, COVID-19, biosafety, and other content. Moreover, the effect of video and online learning is far less than face-to-face teaching.

The results of the semi-open question collection are shown (Table 4). Most students described their autonomous learning ability had been fully exercised, and they have become better aware of their professional work. Meanwhile, teachers agreed with the new teaching mode of self-study, questioning, and guidance and phased assessment facilitated students’ learning. Regarding disadvantages, some students thought that the self-study tasks and frequent groups assigned by teachers would make them feel overburdened, and they often spent more time on in-depth study. Teachers believed the current teaching syllabus did not cover enough knowledge points, and more time and energy would be required to design teaching plans and record and develop video courses.

**Table 4:** According to teachers and students, the advantages and disadvantages of teaching work in the post-epidemic era.

Student	%α	Teacher	%α
**Advantages**			
Improve autonomous learning ability	66	The new teaching model is complete in points	42
Deepen the understanding of TDM work	58	Epidemic prevention and control have improved	33
Broaden research thinking	26	Regular assessment can timely understand the learning situation of students	25
Gain more clinical expertise	26	Students familiarize themselves with the courses and improve the quality of on-site teaching	17
Knowledge points from shallow to deep, easy to understand	11	Avoid task-based test schedules	8
**Deficiencies**			
Self-study overload	37	The syllabus is not perfect enough	33
Frequent study reports bring a sense of stress	21	It takes more time and effort to design lesson plans	33
Fewer theoretical courses	13	More time is required to participate in the recording and programming of video courses	17
Low efficiency of self-study	13	Not every student can study by himself seriously	8
sense of distance	5	In the early stage of teaching, students lack the opportunity for field experiment operation	8

α The proportion of semi-open answers mentioned by teachers and students.

### 3.2. Effect evaluation of biosafety training

**Figure 3.**
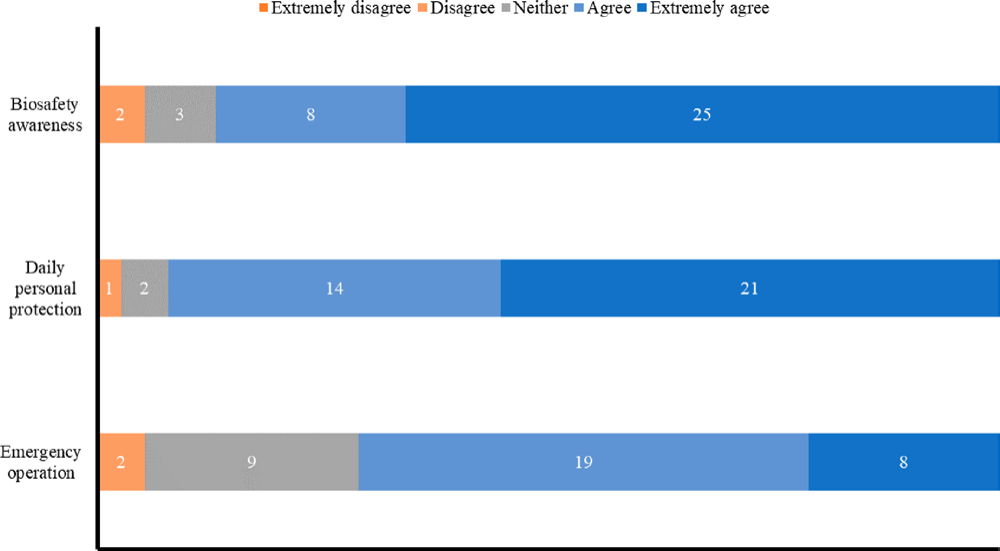
Results of students’ evaluation based on the biosafety training effect.

**Figure 4.**
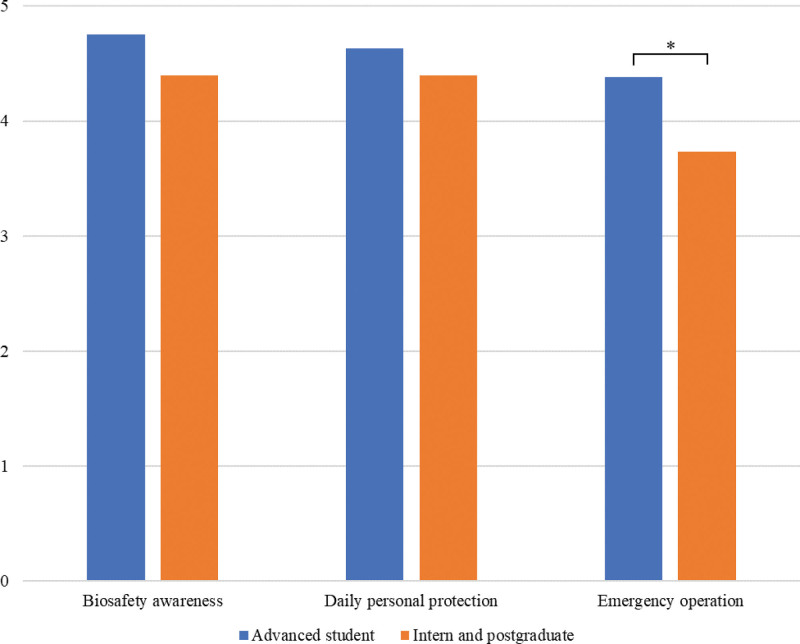
Comparison of the evaluation results of biosafety training effect among advanced students, interns, and graduate students. Numerical value is the average of the analyzed items. All data have passed the normal distribution and homogeneity test of variance and were compared using the independent sample *t* test, *P < .05.

No students chose the 1-point option in the biological safety training effect evaluation (questions 11–13) (Fig. 3). Biological safety consciousness was significantly higher in 25 students (65.8%) after lab access training. Moreover, 21 students strictly followed the management system of the laboratory biosafety of daily demand (55.3%). However, only 8 students thought they could handle the emergent biosafety problems perfectly (21.1%). Combined with the analysis of questionnaire results of 3 groups of students (Fig. 4), the self-assessment of advanced students was higher than that of interns and postgraduates (4.38 vs 3.73, *P* < .05). The reason is that the students did not participate in the rotation learning in the pharmacy department, providing them with more time for practical training.

### 3.3. Effect evaluation of unique teaching methods in the post-epidemic era

**Figure 5.**
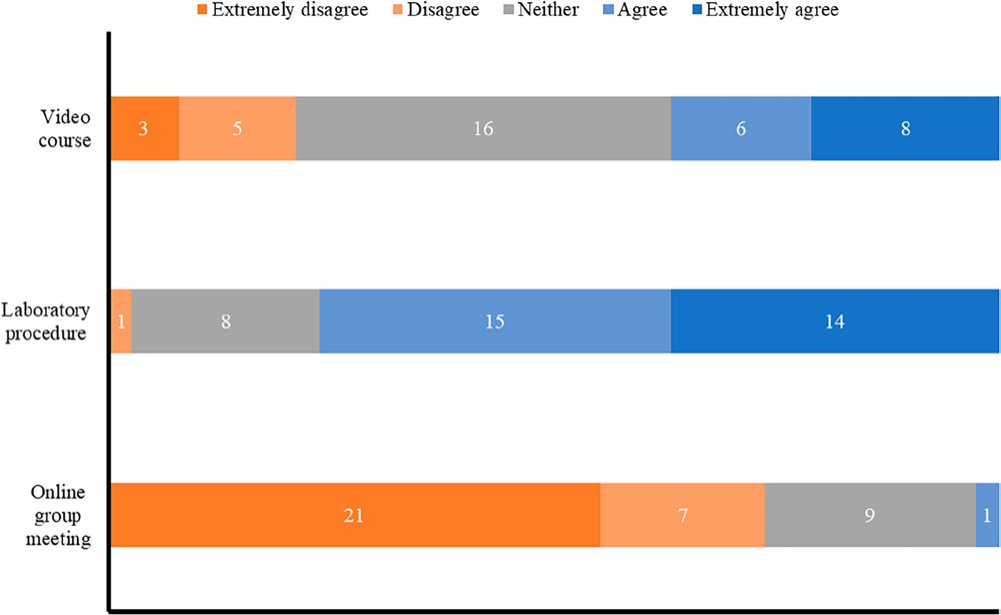
Results of students’ evaluation of the effects of unique teaching methods in the Post-Epidemic Era.

**Figure 6.**
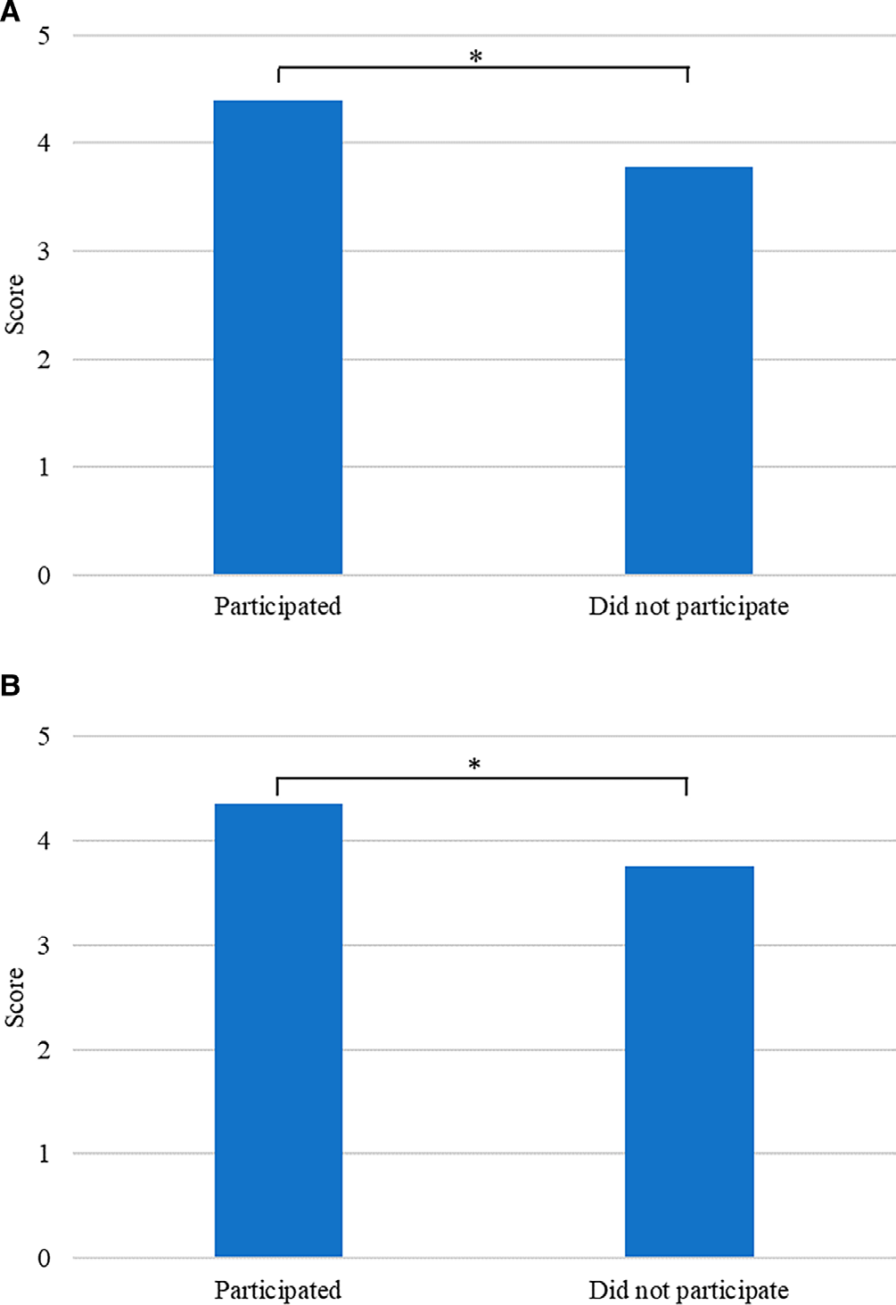
Comparison of the online guidance between students who have participated in the new project development or research and those who did not participate. (A) Comparison of the online guidance evaluation results of teachers by students who have participated and not participated in the new project development. (B) Comparison of online guidance evaluation results of teachers by students who have participated and not participated in the project research. The values have been expressed as the average of the analyzed items. All data have passed the normal distribution and homogeneity test of variance, and were compared through the independent sample *t* test, *P < .05.

Students’ attitude mainly towards video courses is neutral (Fig. 5). 29 students say that teachers can provide satisfactory experimental guidance online but prefer face-to-face discussions with teachers and classmates in the laboratory (76.3%). 20 students developed the new testing items, and 22 participated in TDM-related research. They communicated with teachers more frequently. Based on the survey results (Fig. 6), they were more satisfied with the online guidance of teachers than other students (4.40 vs 3.78, *P* < .05; 4.36 vs 3.75, *P* < .05).

### 3.4. Students’ self-perception of professional ability improvement

**Figure 7.**
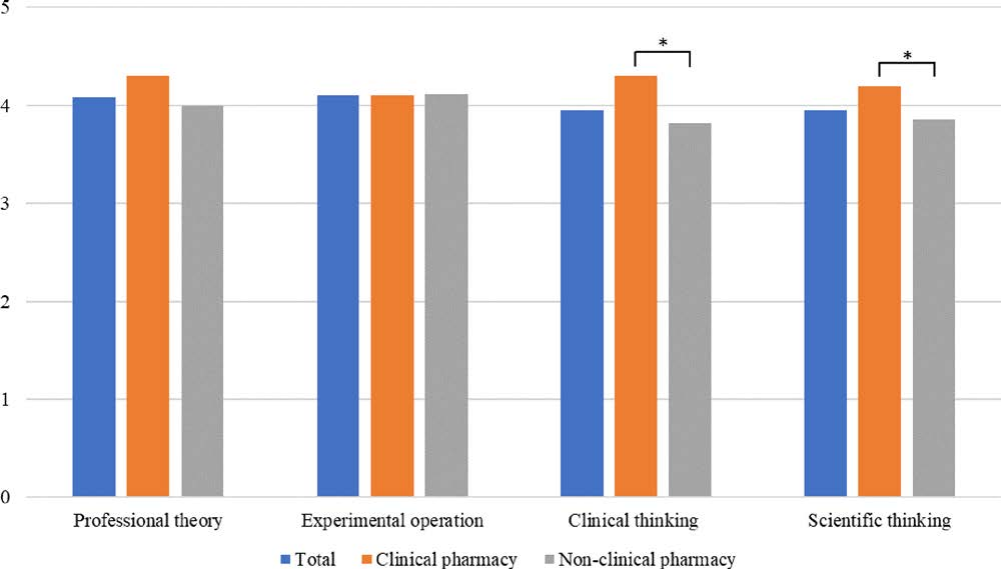
Comparison of self-perception results of students on the improvement degree of professional ability. Numerical value is the average of the analyzed items. All data have passed the normal distribution and homogeneity test of variance and the independent sample *t* test compared to different samples, *P < .05.

Apart from the short-term graduates and 2 interns who dropped out due to personal reasons, 34 students participated and passed the completion examination with an average score of 76.17 (over 60 acceptable, over 80 excellent). The assessment results can be more intuitive to evaluate the training improvement effect. However, after improving teaching work, the survey object was the first class of students. They accepted the new teaching and assessment content, and we could not make a direct comparison with the results of the past student assessment. Some teachers suggested that students evaluate their improvement in 4 aspects to solve this problem: professional theory, practical operation, clinical thinking, and scientific research ability. The results were expected (Fig. 7). Students generally believed that their professional skills were improved after learning and influenced by the direction of their major. The self-perception level among clinical pharmacy students was higher than that of non-clinical pharmacy students based on clinical thinking and scientific research ability.

## 4. Discussion

The global epidemic outbreak has alarmed laboratory researchers. Although China has made significant achievements in epidemic prevention and control, minimizing the risk of virus transmission, routine biosafety protection in laboratories is still a priority that is hard to ignore. We have established a “progressive” teaching system through questioning, guidance, and other teaching methods, cultivated students’ independent learning ability from simple to profound, adopted stage assessment to dynamically detect students’ learning quality, and strengthened communication between the teachers and students.

The mainstream theory teaching method is given priority to teachers’ teaching. Its advantage is that teachers can use introverted students to output enough knowledge in a short time. However, students can only passively take notes in class without thinking independently, cooperative learning, and lifelong learning ability, which decreases the enthusiasm of the class.^[16]^ In recent years, many new teaching modes different from traditional teaching have emerged, including simulation scenario teaching,^[17]^ team-based teaching,^[18]^ problem-oriented teaching,^[19]^ and case-based teaching.^[20]^ It aimed to cultivate students’ autonomous learning ability and improve their comprehensive quality. However, we should not wholly abandon traditional theoretical teaching but combine it with the modern teaching model.

We should further optimize the teaching syllabus, avoid assigning excessive learning tasks to students and establish a system of rewards and punishments to encourage teachers to design good curriculum content. Regarding laboratory access training, we will increase the number of training hours and carry out practical courses depicting the negative textbook on biosafety operation. Every student will accumulate experience in dealing with emergencies through trial and error on the premise that safety is guaranteed. This study uses video courses and online learning/group ways. Simultaneously, reducing staff assembled offline breaks the space constraints. However, the boring video learning process makes students weary, reduces self-study motivation, and sometimes, due to network instability and environmental noise of hard-line, the quality of teaching decreases. Teachers should reduce the duration of explaining theoretical knowledge in video courses and carry out face-to-face teaching depending on the epidemic prevention and control policies.

Since this is the first study of teaching improvement attempts during the epidemic, we know that changing teaching forms will bring a series of unpredictable difficulties. However, teachers and students comment more positively on the teaching improvement, and students generally believe that their professional level and learning ability have improved after participating in learning. In the future, the institute will continue to establish a new teaching form to carry out the teaching work, especially the biological security problem, to undergo prevention and control as routine work. Feedback suggestions from teachers and students ensure continuous optimization. Therefore, this research inspires other teaching laboratories or those interested in constructing teaching laboratories or institutions.

## 5. Conclusion

In the post-epidemic Era, a laboratory teaching system based on online guidance and offline self-study was established. Teachers used online lectures, video courses, and other forms to help students gather biosafety and professional theoretical knowledge and provide experimental guidance for students through various online platforms. The teaching methods based on questioning and advice can help students improve their independent learning abilities, broaden their research thinking, and maintain full communication between teachers and students during the learning process. Evaluations and suggestions of teachers and students on teaching work assist our shortcomings in achieving a preferable teaching effect. This study provides novel ideas for laboratory teaching in the post-epidemic period by optimizing the faculty structure, strengthening offline personnel management, and reasonably exploring online teaching modes.

## Acknowledgments

The authors are deeply grateful to all teachers and students who participated in our research gave very detailed feedback, and especially to those who participated in the survey and gave very detailed feedback that allowed us to critically evaluate our teaching. The authors would also like to thank Xiangya Hospital of Central South University for their support to our work.

## Author contributions

**Conceptualization:** Qi Huang, Qiong Liu, Yueping Jiang.

**Data curation:** Qi Huang, Hong Su, Yingfan Zhang, Qiong Liu, Yueping Jiang.

**Formal analysis:** Qi Huang, Hong Su, Yingfan Zhang, Qiong Liu, Yueping Jiang.

**Investigation:** Qi Huang, Hong Su, Qiong Liu, Yueping Jiang.

**Methodology:** Qi Huang, Hong Su, Qiong Liu, Yueping Jiang.

**Project administration:** Qi Huang, Shao Liu, Qiong Liu, Yueping Jiang.

**Resources:** Qi Huang, Shao Liu, Qiong Liu, Yueping Jiang.

**Software:** Qi Huang, Hong Su, Shao Liu, Qiong Liu, Yueping Jiang.

**Supervision:** Qi Huang, Qiong Liu, Yueping Jiang.

**Validation:** Qi Huang, Yingfan Zhang, Qiong Liu, Yueping Jiang.

**Visualization:** Qi Huang, Yingfan Zhang, Qiong Liu, Yueping Jiang.

**Writing – original draft:** Qi Huang, Hong Su.

**Writing – review & editing:** Qiong Liu, Yueping Jiang.

## Supplementary Material


