# Supplementary material for: Practice and experience in the teaching system of clinical pharmacy laboratory in the post-epidemic era: A review
Source: Medicine (Baltimore). 2022 Dec 9;101(49):e32223. doi: 10.1097/MD.0000000000032223 (PMC9750525; doi:10.1097/MD.0000000000032223)
Supplement: Supplementary file 1 [file medi-101-e32223-s001.pdf]

Table S1 Clinical pharmacy laboratory biosafety level ii/III personal protective equipment wear and doff operation assessment standards[a].

student's name:_________ rank examination: Level 2 / Level 3 _ Score:________

project	Score points	Score	Mark
**Preparation stage**		**2**	
1. Prepare yourself	Wear suitable work clothes, work shoes, no long fingernails, and remove personal belongings (jewelry, watch, etc.).	1	
2. Goods ready	Prepare with: the N95 respirator, waterproof rubber or shoe cover, shoe covers, protective clothing, disposable working cap, latex gloves, goggles/protective face screen, comprehensive type of respiratory protection device or a higher level of electric supply air filter respirator, disposable medical gowns, quick-drying hand sanitizers, choose a suitable model, check with the effect of content and packaging integrity.	1	
**Wear personal protective equipment**		**34**	
1. Hand hygiene	Follow the "seven-step wash technique" for hand hygiene.	2	
2. Wear a medical	To check the integrity of the mask, support the protective mask with one hand, nose clip up, and support	1	

protective mask (or N95 mask)	the mask under the chin.	
	Put on the mask: first, pull down the belt on the neck, then pull up the belt on the middle of the head, and adjust the belt.	2
	Start with your fingertips in the middle and move sideways to press the nose clip to shape the bridge of your nose.	3
	Air tightness check: no air leakage from the mask.	2
3. Wear a disposable work cap	The elastic side of the hat is pulled back from the forehead to the back of the head so that the hair is not exposed.	2
4. Wear protective clothing	Check whether protective clothing is damaged and that the zipper is in good condition.	2
	Hold the cap and sleeve of protective clothing in your hands and avoid touching the ground.	3
	First, put on the clothes, then put on the jacket, zip up to the chest, then buckle the protective cap to the head. The disposable work cap must cover the protective clothing and, finally, zip up to the top.	4
	After the zipper is completely closed, tear and flatten the sealing tape to cover the zipper and check the tightness of the joint between the mask and protective clothing.	3
5. Wear gloves	Check gloves for air tightness.	1
	Wear gloves and cover the cuffs of the protective suit with gloves turned over.	3

6. Wear goggles/protective face screen	Check the elastic band of the goggles (protective face screen) and wear the goggles (protective face screen). Adjust the comfort level, and make the lower edge of the glasses and the mask as close as possible (the upper edge of the face screen is pressed against the brim of the protective suit) without exposing the face.	2
7. Wear disposable waterproof boot covers	Wear disposable waterproof boot covers and insert the bottoms of protective clothing into the boot covers.	2
8. Check for stretch and tightness.	The operator does hand lifting, body turning, leg lifting, squatting, bending, and other actions to check whether the protective clothing fits appropriately and interferes with the operation.	1
	Please dress strictly for peer or mirror inspection (oral).	1
**Remove personal protective equipment**		**50**
1. Hand hygiene	Strictly follow six-step hand washing.	2
2. Remove the goggles/protective face screen	Remove the elastic band from the top of your head with both hands, and remove the goggles by tilting your head forward. Put the disposable into the medical waste bucket and the reused into the designated recycling container. Hand hygiene (six steps to hand washing).	5
3. Take off the	Gently unfasten the sealing tape and pull the zipper to the end without touching the protective mask.	5

protective clothing	Pull the cap up and away from the head.	5
	Take off the sleeves, dirty face inwards, from top to bottom take off the side roll.	10
	Remove clothing and gently roll it up to the ankles with contamination facing inward. Remove waterproof boot covers (and gloves) together with protective clothing and place them in the clinical waste bucket. Hand hygiene (seven steps for hand washing).	10
4. Unglove	Remove gloves (if not all gloves in step 3). Hands do not touch other parts, such as work clothes and hand hygiene (seven steps to wash hands).	4
5. Remove the disposable work cap	Reach the inside edge of the hat behind the ears with both hands, remove the hat from the inside, wrap the hat with the reverse side, and put it into the medical waste bag with hand hygiene (seven steps of hand washing).	4
6. Remove medical protective masks	Lower your head slightly, take off the straps first, then take them off, hold them and put them into the medical waste bucket, and wash your hands (seven steps).	5
**Effectiveness evaluation**		**14**
1. Operation time: 20 minutes	One point will be deducted for more than 5 minutes.	2
2. Effectiveness	Operation specification; Skilled procedure; The action is light and steady.	4

evaluation		
	Dress tightly without showing skin.	2
	When removing protective equipment, hands do not touch the face and other bare skin and mucous membranes.	2
	When removing protective clothing, the contaminated surface will be completely involved on the inside, clean side outwards.	2
	Do not touch clean surfaces with your hands when taking off protective clothing.	2

[a]When removing personal protective equipment, perform hand hygiene at each step. If there is no visible pollution on the wrist from Steps 1 to 4, the six-step washing method is recommended to prevent the sleeve from soaking; Steps 5 to 7 are the seven-step washing techniques. Six-step (or seven-step) points deduction requirements for washing techniques: if there is an omission, it will be judged as failing the assessment; Missing steps will deduct 1 point from each step; If the rubbing strength is not enough, or the rubbing time is not in place, 1 point will be deducted from each time. Level 3 Protection In addition to the above protection requirements, goggles (anti-fog type) or protective face screen should be replaced with a comprehensive respirator or higher level respirator with an electric air supply filter (positive pressure type head cover), protective clothing, and a layer of disposable medical isolation clothing.
