# Supplementary material for: Practice and experience in the teaching system of clinical pharmacy laboratory in the post-epidemic era: A review
Source: Medicine (Baltimore). 2022 Dec 9;101(49):e32223. doi: 10.1097/MD.0000000000032223 (PMC9750525; doi:10.1097/MD.0000000000032223)
Supplement: Supplementary file 2 [file medi-101-e32223-s002.pdf]

Table S2 Laboratory assessment standards for simulating blood samples from patients with confirmed or suspected COVID[a].

Student’s name:_________ score:________

Item	Score points	Score	Mark
**Preparatory phase**		**5**	
1. Prepare Yourself	Biosafety level 3 personal protective equipment (PPE) has been adequately worn according to standards.	1	
2. Goods ready	Simulated blood sample, transport box, biosafety tank, sealed bag, 75% ethanol, chlorine disinfectant, watering can, medical rag, centrifuge, scroll machine, pipette gun, medical absorbent pad, hydrogen peroxide disinfectant, 0.9% sodium chloride solution, yellow medical waste bag.	2	
3. Oral expression	Samples from confirmed or suspected patients have dressed in biosafety level-iii personal protective equipment during the laboratory operation, and the laboratory environment met the requirements of Level-II or above biosafety laboratory.	2	
**Samples received**		**15**	
1. Oral expression	Specimens of patients confirmed or suspected by the transport box, biological safety cans and a double sealed bag of three layers of container transport, transport box with a unified on biological hazards identification, have been confirmed in the link of disinfection before packing. The quantity of samples checked sample name, gender, age, number, and testing project information such as the sign said when	3	

	confirmed receiving.	
2. Operation requirement	Before unpacking, use 75% ethanol spray to sterilize the surface, and immediately after unpacking, use 75% ethanol spray to sterilize the surface and remove the biosafety tank.	4
	In the biosafety cabinet, remove the double-sealed bag from the biosafety tank and disinfect it with 75% ethanol spray.	4
	The blood sample is removed from a double-sealed bag and disinfected with a 75% ethanol spray in a biosafety cabinet.	4
**Sample inactivation operation**		**5**
1. Oral expression	For the thermal stability samples, the inactivation treatment was carried out by heating the samples with a water bath at 65℃ for 20 ~ 30min. The samples could be treated with short-wave ultraviolet radiation if conditions were available for the thermal instability samples.	5
**Sample pretreatment**		**45**
1. Oral expression	All pre-sample processing operations are completed in a Class 2A and above biosafety cabinet.	1
2. Operation requirement	Before the experiment, wipe the biosafety cabinet with 75% ethanol and do UV disinfection.	4
	Before centrifugation or vorticity, check whether the collection vessel or EP tube is damaged, whether	4

	the cap is tight, etc.	
	When the specimen is centrifuged, the operator should not leave the centrifuge.	4
	No accident occurred when the specimen was centrifuged. The centrifugation was stopped for more than 10 minutes, and the cover was opened for disinfection with 75% ethanol spray.	6
	Open the tube cover gently and slowly, keeping a distance from the operator's face to minimize the duration of opening.	10
	When using the pipetting gun, the biosafety counter is covered with a layer of disposable absorbent material soaked in disinfectant to prevent samples from dripping onto the table and causing contamination.	4
	The nozzle should stick to the wall during pipetting and slowly blow out the reagent or sample to avoid aerosol generation.	6
	No wrong operation of stirring, pumping, and repeated suction occurred.	6
**Test sample**		**5**
1. Oral expression	According to the specific use, 75% ethanol and chlorine disinfectant can be used to clean the probe and pipeline of the instrument.	2
2. Operation requirement	Before injection, check whether the injection bottle is damaged. The operation should be gentle and	3

	careful to prevent the sample from spilling due to dumping the injection bottle.	
**Handling of laboratory biosafety errors or accidents**		**25**
1. Simulation drilling	The handling of contaminated and damaged personal protective equipment.	5
	The management of occupational exposure (skin, mucous membrane).	5
	Disposal of the spilled blood samples (restricted contamination, laboratory contamination).	5
	Handling of centrifugal accidents.	5
	Process operation of registration and reporting.	5

[a]Before and after each step and the operation of the biosafety cabinet, hand hygiene should be done based on the requirements of the "six-step washing technique." If there is any omission, it will be judged as failing the assessment; Missing steps will deduct 1 point from each step; If the rubbing strength is not enough, or the rubbing time is not in place, 1 point will be deducted from each time.
